# A Remote-Sensing Ecological Index Approach for Restoration Assessment of Rare-Earth Elements Mining

**DOI:** 10.1155/2022/5335419

**Published:** 2022-07-14

**Authors:** Huichao Hao, Zeke Lian, Jing Zhao, Hesong Wang, Zhechen He

**Affiliations:** ^1^College of Landscape Architecture, Beijing Forestry University, Beijing 100083, China; ^2^School of Ecology and Nature Conservation, Beijing Forestry University, Beijing 100083, China; ^3^Beijing Forestry University, Beijing 100083, China

## Abstract

In order to meet the requirements for comprehensive and multidimensional generalization of ecological management effectiveness evaluation indexes in the context of ecological restoration advocating comprehensive management by multiple means, this paper explores the rationality of using RSEI as an ecological management effectiveness evaluation index to adapt to the systematic transformation of the management goal of abandoned mine restoration from ecological restoration to regional socioeconomic sustainable development. Based on Landsat-8 image data, the remote sensing ecological index (RSEI) was used to evaluate the dynamic changes and spatial and temporal differences of the ecological environment in the study area under the long-term multimeans comprehensive management. The RSEI is suitable for evaluating the effectiveness of comprehensive ecological management in mining areas with a large amount of bare soil. The regional RSEI mean value increased by 0.029 in the early stage and 0.051 in the later stage by fragmentation management, indicating a better effect of multimeans comprehensive management. The remote sensing ecological index can objectively reflect the difference of spatial distribution characteristics of ecological environment in the four “Ecological+” governance regions. It can both objectively reflect the ecological status of the study area and reflect the differentiated spatial distribution characteristics of the ecological environment in different treatment areas, which is of long-term practical significance to the ecological construction of the study area. This study provides a theoretical reference for ecological assessment of complex situation under difficult site conditions.

## 1. Introduction

The interaction of ecological protection and economic growth is an important research topic worldwide [1], for maintaining a balance between the ecoenvironment and the economy which will enhance the well-being of humanity [2]. The ecological restoration of mines is a comprehensive problem coupled with multiple elements such as energy, ecology, land use, and landscape. The management objectives and means of abandoned mine restoration are undergoing a systematic transformation [3–5] from ecological restoration [6] to sustainable regional socioeconomic development [7, 8]. With the improvement of mine ecological restoration technology, the diversification of treatment means and the transformation of treatment ideas and treatment goals have put forward higher requirements for the evaluation of mine treatment effectiveness: the ability to take into account the characteristics of long time span and large spatial scale of mine ecological restoration, while being able to comprehensively evaluate the treatment and restoration of abandoned mines with diverse means and goals, has become the focus of relevant research.

The detailed assessment for the sample sites [9–13] provides data results limited to local spatial heterogeneity, making it difficult to form a comprehensive global evaluation, because it can only be based on physical and chemical studies constrained by spatial and temporal conditions. At the macrolevel, remote sensing technology, with its multitemporal, high spatial coverage and easy and rapid access, has become an important tool for comprehensive evaluation of mine management effectiveness [14–17]. However, most of the related work is based on basic observation using a single index [18], among which vegetation indexes are especially common [19], while the sensitivity of vegetation index is low. For abandoned mines with serious ecological damage and bare soil and rock, it is difficult to reflect the slight difference of treatment effectiveness only from the perspective of vegetation and it is even more difficult to make a comprehensive evaluation taking into account the special characteristics of ecological restoration with multiple means and multiple objectives.

In 2013, Xu Hanqiu proposed remote sensing ecological index (RSEI) as a monitoring and evaluation tool for the regional ecological condition [20], which is a rapid detection and evaluation of the regional ecological long time series [21]. Among the four ecological factors of remote sensing ecological index, the heat index and dryness index obtained by the participation of surface temperature and bare soil index in the inversion reflect a high contribution rate in the evaluation of ecological quality for mines and are closely related to human life [22–24], which has a more comprehensive and social standard applicability and makes up for the shortcomings of the traditional evaluation method of using normalized vegetation index to evaluate ecological quality. A large number of existing studies have been conducted on the evaluation of ecological conditions in multiscale and multitrait areas such as cities and urban agglomerations [25–29], nature reserves [30–33], soil erosion areas [34–36], and mines [37] based on the RSEI, but few discussions related to the applicability and limitations of the assessment methods of different mine management tools have been found.

The ecological management process of abandoned mines in Xunwu County is a typical representative of the change in thinking about the management of abandoned mine land in China in recent years. Local governance has shifted from a focus on a single ecological issue to one of sustainable regional economic and social development. The assessment of its ecological governance effectiveness needs to be made through an objective ecological index to make a long time series and multidimensional generalized evaluation of its two-phase and multimethod ecological governance from 2013 to 2019. This paper selects abandoned mines in Xunwu County as the study area and aims to answer the following questions: (1) Is there any difference in the effectiveness of ecological management between the two objectives of “plant regreening” and “sustainable regional economic and social development”? (2) What are the similarities and differences in the spatial and temporal dynamics of the ecological environment of each quality level within the scope of different ecological management tools? (3) Is the remote sensing ecological index (RSEI) applicable to the assessment of the effectiveness of regional ecological management in the complex situation of long-term multimethod integrated management? This study is expected to enrich the evaluation system of ecological restoration effectiveness in mining areas and also has long-term practical significance for ecological construction in the study area.

## 2. Study Area

Xunwu County, Ganzhou City, Jiangxi Province, is located at the junction of Gan, Guangdong, and Fujian Provinces and belongs to the key ecological function area of forest biodiversity in the South Ridge Mountains, which is an important part of the southern ecological barrier. Xunwu County is rich in rare-earth resources and is one of the earliest counties in China to mine rare earths. After more than 30 years of mining, there are more than one hundred mining mineralization points left [38]. Because of the backward production process, sloppy management mode, a large number of pool leaching, heap leaching, and *in situ* leaching, and other destructive mining means, the mine remains in the destruction of vegetation, soil erosion, river siltation, flooding of arable land, water pollution, soil acidification, and other ecological problems, a large area of the mine surface layer of humus layer is lost, mining area strong weathering layer or semiweathering layer is exposed, and the ecological environment around the mine is also constantly degraded.

Since 2010, the local area began to pay attention to the multiple ecological problems brought about by abandoned mines and made a long and extensive attempt. In the current perspective, its ecological management work can be divided into two stages: from 2013 to 2016, Xunwu County adopted the objective of “revegetation” and adopted the way of land remediation and revegetation by dividing the sites in phases to manage many abandoned mining areas in the county, but the results were not good; in 2016–2019, taking the wisdom of the previous management experience, Xunwu County, in view of the characteristics of different rare-earth mining areas [39], took “regional economic and social sustainable development” as the target, summarized “Ecology + Industry,” “Ecology + Photovoltaic,” “Ecology + Poverty Alleviation,” and “Ecology + Tourism,” the four means of comprehensive management of development, in accordance with local conditions, and finally achieved significant management results.

**Figure 1:**
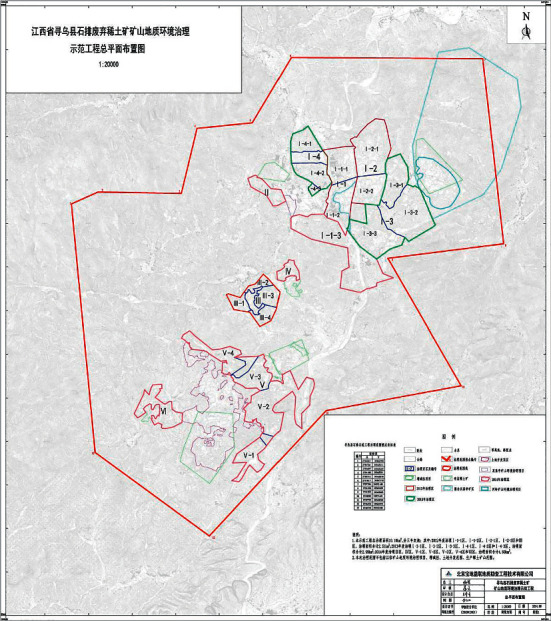
General layout of Xunwu abandoned rare earth mine geological environment control demonstration project (left).

**Figure 2:**
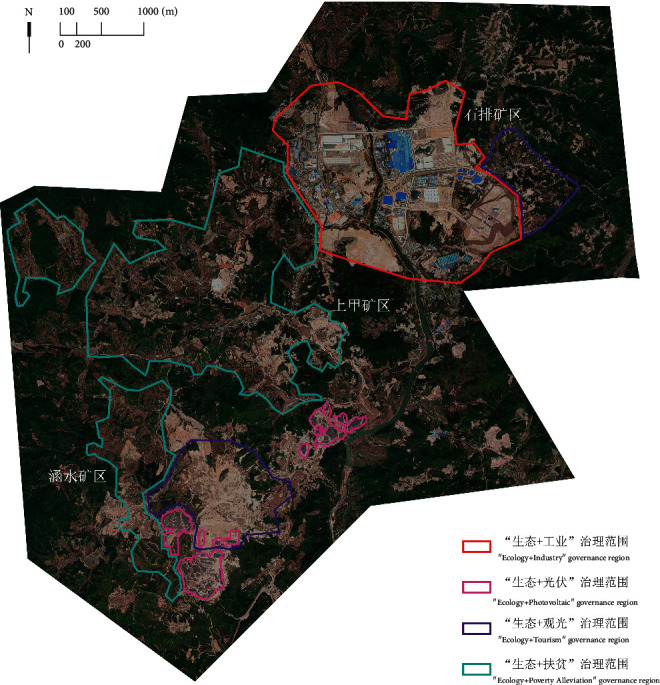
Schematic diagram of four “Ecological+” governance regions (right).

The study selected the area delineated in the Environmental Impact Report of the Construction Project of the “Demonstration Project of Geological Environmental Treatment of Abandoned Rare Earth Mines in Shipai, Xunwu County,” which was implemented from 2013 to the end of 2015 [40], as the study area, with a total area of 224.7264 km^2^ (Figure 1). After 2016, Xunwu County carried out comprehensive zoning treatment and four kinds of “Ecology+” treatment methods were implemented in the study area. Based on the 2019–2035 plan, 2013–2020 remote sensing satellite images, and other information, the treatment scope of the four treatment measures was divided and selected (Figure 2) to reflect the treatment effectiveness of different treatment tools.

The “Ecology + Industry” governance region is located in the northeast side of the Shipai mining area. The presence of an industrial park in the area could solve both the environmental problems and industrial platform problems. The “Ecology + Photovoltaic” governance region is distributed in the Hanshui governance region on the barren slopes, to generate income for Xuanwu power supply. “Ecology + Poverty Alleviation” is implemented through the construction of high-standard farmland to plant economic crops around the developed mining area to achieve revegetation and promote local farmers' income, and there is no clear boundary for the governance region. The “Ecology + Tourism” governance region is mainly distributed in the north side of Hanshui mine, using the mining site to enrich the cultural ecology of the mine and strengthen the cultural tourism and leisure industry. The four governance methods are tailored to local conditions, taking into account the regional cultural characteristics of the mining industry and the needs of regional socioeconomic development, to provide a sustainable impetus for the ecological restoration of the whole area of the mine.

## 3. Methods

### 3.1. Remote Sensing Data and Preprocessing

Landsat has a large amount of historical data compared with other satellite data, which is conducive to dynamic monitoring of the ground surface for a long time series [41]. In this paper, Landsat remote sensing images are used as the data source to analyze and evaluate the ecological environment of mines in Xunwu County. The data are obtained from the United States Geological Survey (USGS) and Geospatial Data Cloud [42]. In order to ensure that the surface ecology will not be different due to seasonal differences. The remote sensing data are selected from July to October, when the vegetation growth condition is good, which is conducive to the evaluation study of the ecological quality of mines [29]. In this paper, Landsat8 TM images from October 2013, September 2016, and September 2019 were used. The selected data all meet the characteristics of high data quality and low cloud content, and the image quality meets the research needs. The remote sensing images are preprocessed in ENVI5.3 software for radiometric calibration and atmospheric correction, and finally the remote sensing images are cropped based on the project boundary of the rare earth mine management project in Xunwu County.

### 3.2. Remote Sensing Ecological Index Construction

Remote sensing ecological index (RSEI) is a more mature model in the study of ecological status evaluation [20], which is based entirely on the information of remote sensing image data and uses the inversion of remote sensing image data to obtain four index factors of greenness (NDVI), heatiness (LST), humidity (WET), and dryness (NDBSI).

(1) $$\begin{array}{c} \text{RSEI}=f\left(\text{NDVI},\text{LST},\text{WET},\text{NDBSI}\right)ɛ. \end{array}$$

The normalized differential vegetation index (NDVI), which is commonly used to monitor vegetation growth and vegetation cover, is used to represent the greenness index, which is a comprehensive response to the growth of surface vegetation; the moisture component (WET), which is related to soil moisture in the tassel cap transformation, is used to represent the moisture index, which is a response to the water content of the surface. The WET component of soil moisture in the tassel cap transformation represents the moisture index, which reflects the surface water content and the regional water-heat balance. The land surface temperature (LST), which reflects the energy flow and material exchange of the soil-vegetation-atmosphere system, represents the heat index, which reflects the condition of the uncovered surface and surface damage; normalized differential build up and bare soil index (NDBSI), the index-based built-up index (IBI), and soil index (SI), which together influence the surface dryness, are used as mean values to represent the dryness index, to observe the thermal pollution of the area, and to assess the possibility of natural hazards at the site. The formulae for calculating the index factors are as follows [43]:

(1) The normalized difference vegetation index (NDVI) was used to represent the greenness component (also called greenness index), which was modeled as
  (2) $$\begin{array}{c} \text{NDVI}=\frac{\left(\rho_{\text{nir}}-\rho_{\text{red}}\right)}{\left(\rho_{\text{nir}}+\rho_{\text{red}}\right)}. \end{array}$$
(2) Humidity component WET based on tassel cap variation is sensitive to humidity; *ρ*_*i*_ indicates the spectral reflectance of the corresponding waveband:
  (3) $$\begin{array}{c} \text{WET}=0.1511\rho_{\text{blue}}+0.1973\rho_{\text{green}}+0.3283\rho_{\text{red}}+0.3407\rho_{\text{nir}}-0.7117\rho_{\text{mir}1}-0.4559\rho_{\text{mir}2}. \end{array}$$
(3) The NDBSI is expressed as the average of the exponential building index (IBI) and the bare earth index (SI):
  (4) $$\begin{array}{c} \text{NDBSI}=\frac{\left(\text{IBI}+\text{SI}\right)}{2}. \end{array}$$
(4) The heat index is expressed in terms of surface temperature LST, and the other indices are calibration parameters:
  (5) $$\begin{array}{c} \text{LST}=\frac{T}{\left[1+\left(\lambda T/\rho \right)\text{ln}\varepsilon \right]}. \end{array}$$

The remote sensing ecological indexes coupling the four indicators were constructed. Because of the differences in the values of the indicators, for this, standardization and dimensionless processing are performed, and the calculation formula is

(6) $$\begin{array}{c} NX=\frac{X-X_{\text{min}}}{X_{\text{max}}-X_{\text{min}}}. \end{array}$$

In the formula, NX is the result of the normalization of the index, *X* is the average of the image elements of this index, and X_max and X_min are the maximum and minimum values of the index, respectively.

### 3.3. Principal Component Analysis

In data mining related applications, principal component analysis is one of the most common methods, and its key advantage is that the weights of each indicator are not artificially determined, which can effectively avoid the influence caused by human subjective factors. Principal component analysis is used to integrate the indicators of remote sensing ecological index, and the principal component transformation is carried out by using ENVI software. The initial value RSEI_0_ is obtained by subtracting PC1 from 1, and then it is standardized. The resulting RSEI is the remote sensing ecological index, calculated by the following formula:

(7) $$\begin{array}{c} \text{RSEI}=\frac{{\text{RSEI}}_{0}-{\text{RSEI}}_{0\_\min}}{{\text{RSEI}}_{0\_\max}-{\text{RSEI}}_{0\_\min}}. \end{array}$$

In the formula, RSEI_0_ is the initial remote sensing ecological index, RSEI_0_max_ and RSEI_0_min_ are the maximum and minimum values of the initial remote sensing ecological index, respectively, and RSEI is the final remote sensing ecological index with the value range of [0, 1]; the larger its value, the better the ecological environment quality.

## 4. Results

### 4.1. Results of Principal Component Analysis

According to the results of the principal component analysis of the four subindicators of the remote sensing ecological index (Table 1), the first principal component PC1 has the highest contribution rate of the six-year eigenvalues, and the contribution rate exceeds 82% except in 2020. That is, the first principal component concentrates the characteristic information of the four subindicators to the maximum extent; the contributions of the four ecological factors to PC1 are relatively stable: WET and NDVI are both positive, mainly reflecting the influence of surface vegetation on the ecological environment, i.e., humidity and vegetation cover play a positive effect on the ecological environment; NDBSI and LST are both negative, mainly reflecting the influence of surface bare soil, construction, etc. on the ecological environment, i.e., the degree of bare ground, man-made construction area, and surface temperature have a negative effect on the ecological environment [43]; this is in line with the general perception of the feedback relationship between the four indicators and the ecological environment quality. Therefore, the first principal component PC1 was used to construct the remote sensing ecological index (RSEI).

Based on the first principal component loadings, the dynamic changes of the first principal component four ecological factors' index values from 2013 to 2019 were observed. The main features in the study area were bare soil and vegetation, and the trends of both changes were consistent. Among the four indicators, the dryness index (NDBSI) contributed the largest absolute value, followed by the greenness index (NDVI). Also, the dryness index deepened its role between 2013 and 2016 and the contribution of the first principal component dryness index reached −0.804 in 2016; by 2019, the contribution of dryness to the area weakened to −0.661, indicating that the comprehensive management after 2016 effectively eliminated the bare soil area in the mine area. The dryness index coupled with the construction index and bare soil index has the maximum contribution in the rare earth mining area, which is consistent with the objective special situation that there is a large area of bare soil in the rare earth mining area [44]; it objectively reflects the applicability of RSEI in the evaluation work of the effectiveness of comprehensive ecological management of mines with a large area of bare soil.

### 4.2. Dynamic Changes in Ecological Quality in the Study Area at Two Stages

**Figure 3:**
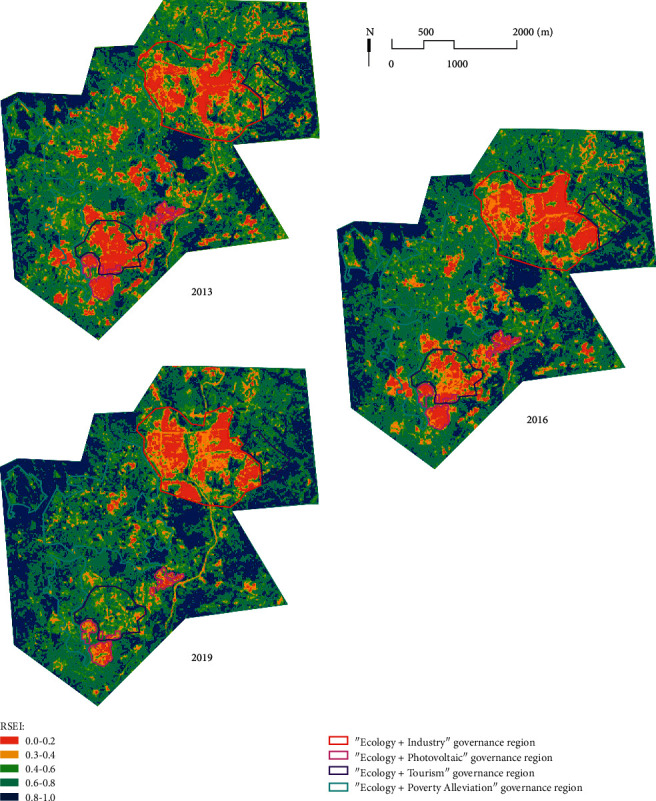
Remote sensing ecological index changes of Xunwu from 2013 to 2019.

When creating the remote sensing ecological index, Xu Hanqiu assigned a uniform gradient color to the areas corresponding to the regional RSEI of 0-1 to visualize the spatial variation of the regional ecological quality. He further divided the remote sensing ecological index into five levels at the interval of 0.2 and examined the rationality of the RSEI by examining the trends of the four indicators among the levels of ecological conditions [25]. This paper refers to this grading and color assignment to further compare the temporal changes of regional ecological quality in similar ecological quality classes. The RSEI values were divided into five intervals of [0, 0.2], [0.2, 0.4], [0.4, 0.6], [0.6, 0.8], and [0.8, 1.0] with equal intervals [25] and graded and assigned color visualization and evaluated as very poor, poor, moderate, good, and very good in order (Figure 3). Also, the area and weight changes of each quality level ecological environment in the study area were counted (Table 2).

The remote sensing ecological indices of the study area before and after the treatment were 0.584 and 0.667, respectively, indicating an overall improvement in ecological quality in the region after the treatment. Comparing the regional ecological quality in 2013 and 2019, the area of areas with poor and very poor ecological quality grades decreased by 7.76% and the area of areas with good and very good ecological quality grades increased by 15%. The areas with very poor ecological environment are concentrated in the mining area, and the overall trend of “peripheral ecological immersion recovery and internal delayed slow recovery” is observed in the whole process of ecological treatment from 2013 to 2019. This trend is most obvious in the mining area of Hanshui District in the southwest of the governance region, reflecting the radiation damage to the surrounding ecological environment caused by mining in the study area and the gradual implementation of long-term treatment work. By 2019, the contiguous low ecological quality areas in Shangjia and Hanshui districts have been largely eliminated; the ecological quality of the Shibai industrial park is poor.

Analyzing the changes in the distribution of ecological grades in the study area in two stages, namely, the prestrip segmentation treatment and the postzone comprehensive treatment, the increase in remote sensing ecological index was 0.029 from 2013 to 2016, while the increase in remote sensing ecological index reached 0.051 after 2016, indicating that the improvement effect of the ecological environment was significantly stronger in the poststage treatment than in the prestage. From the change of area of each quality grade, the area of areas with poor and very poor quality grades only decreased by 2.37% during 2013–2016, and the shift of ecological quality grades occurred mainly for the transformation of areas with medium-quality grades into good grades; such areas were distributed around the periphery of Shangjia and Hanshui mines and mining areas, while the ecological quality of mining areas in Shibai District continued to degrade. During 2016–2019, nearly 10% of the regional ecological quality grade was improved to excellent, indicating that the later comprehensive management improved the regional ecological quality on a large scale.

### 4.3. Analysis of Changes in Ecological Quality in Different Governance Regions

**Figure 4:**
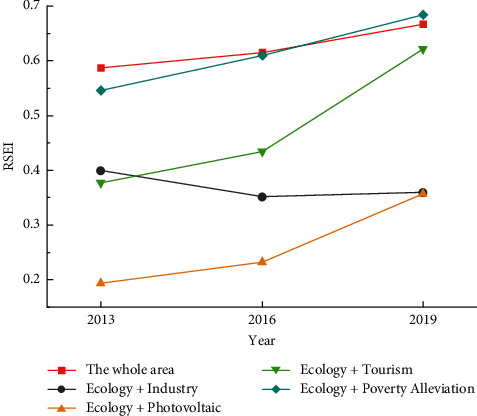
Remote sensing ecological index change curve of different governance areas and overall area.

In order to further compare and analyze the effectiveness of the four “Eco+” management tools, the remote sensing ecological index values of the four designated “Eco+” management areas were calculated (Table 3) and the corresponding line graphs (Figure 4) were drawn to reflect the ecological quality within the areas. The corresponding line graphs (Figure 4) reflect the degree of change of ecological quality within the regions and the influence of each region on the ecological quality of the whole study area.

From the changes of ecological quality, the ecological quality of the “Ecology + Poverty Alleviation,” “Ecology + Photovoltaic,” and “Ecology + Tourism” governance regions has been continuously improved, and the ecological quality of the two governance regions has been improved in the process of the two phases. The ecological quality of the areas treated by “Ecology + Poverty Alleviation,” “Ecology + Photovoltaic,” and “Ecology + Tourism” continues to improve, and in the two stages of treatment, the ecological improvement is greater in the later stage of comprehensive treatment. The RSEI of the “Ecology + Poverty Alleviation” governance region improved by about 0.062 in the early stage and 0.077 in the later stage, and the ecological quality as a whole kept improving at a uniform rate. Only the ecological quality of the “Ecology + Industry” governance region showed a general trend of degradation, but the rate of decline in the later stage was significantly slowed down. In terms of ecological quality, the “Ecology + Poverty Alleviation” governance region always maintains the best before, during and after the remediation process, and plays a positive role in the overall ecological environment. In comparison, the ecological quality of “Ecology + Photovoltaic,” “Ecology + Tourism,” and “Ecology + Industry” governance regions has been improved after treatment, but it still has a negative effect on the overall ecological evaluation. The overall ecological quality of the treated areas has been improved after treatment, but it still has a negative effect on the overall ecological evaluation. In terms of the degree of improvement of regional ecological quality by the treatment measures, the ecological quality of the “Ecology + Tourism” governance region has been improved the most and the fastest, with an increase of 0.244 in the RSEI value from 2013 to 2019. The initial RSEI value of the governance region is only the worst 0.173, and even though the final RSEI value is only about 0.357, which is still the lowest value among the four governance regions, the increase is also significant compared with 2013.

**Figure 5:**
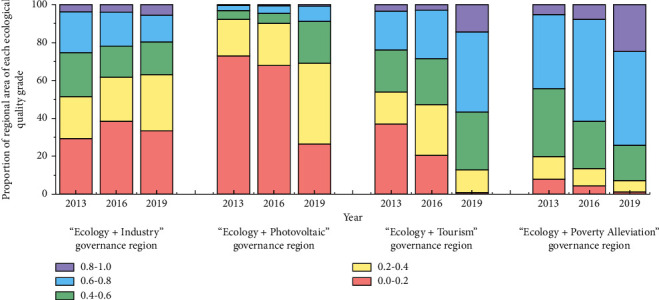
Percentage accumulation chart of ecological environment quality within the four “Ecological+” governance areas.

To further compare and analyze the characteristics of ecological quality changes within the four “Ecology+” governance regions from 2016 to 2019, the percentages of different ecological grade like elements within the scope of different treatment methods implemented in 2013, 2016, and 2019 were counted, and the area of different grades of ecological environment was obtained as a percentage of the total area of percent (Figure 5).

The governance regions of “Ecology + Tourism,” “Ecology + Poverty Alleviation,” and “Ecology + Photovoltaic” all show a trend of continuous conversion of ecological quality from low to high. The “Ecology + Tourism” and “Ecology + Poverty Alleviation” areas have the most obvious improvement on the original bare soil areas of mines, and by 2020, the percentage of areas with very poor ecological quality has been reduced to 0.88% and 1.25%, respectively. In contrast, the ecological restoration of the “Ecology + Poverty Alleviation” governance region is more thorough. The area of the “Ecology + Photovoltaic” governance region with an initial RSEI value of <0.4 accounted for 92.14%, and the area of the same grade was reduced by 22.96% after the solar panels were laid in 2019. The area of the area with very poor ecological quality grade was reduced by the largest amount, 46.53%, and the area of the area with RSEI value > 0.6 increased by 5.84%. The ecological quality of the “Ecology + Industry” governance region has not been improved significantly, but the area of the area with very poor ecological quality has been reduced from 38.55% in 2016 to 33.41% in 2019 and the area of the area with excellent ecological quality has been improved by 1.57%. Compared with the original bare soil of the mine, the construction of the industrial park plays a certain role in ecological improvement.

**Figure 6:**
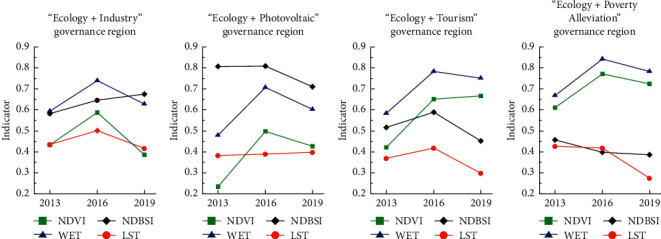
The mean changes of normalized indexes of ecological factors in four “Ecological+” governance regions.

The reasons for the changes in ecological quality within different governance regions were further analyzed based on the changes in the normalized index values of ecological factors in the four regions from 2013 to 2019 (Figure 6). Collectively, the ecological quality status of each region is mainly influenced by vegetation and bare soil area. After 2016, the heat index of the “Ecology + Industry” governance region decreased, which was favorable to the improvement of regional ecological quality, but the greenness and humidity index decreased and the dryness index increased, which reflected the degradation of regional ecological quality due to the decrease of vegetation and the increase of bare soil and construction area in the area. The highest heat index of the “Ecology + Photovoltaic” governance region is in line with the characteristics of its base site, and after 2016, despite the decrease in greenness and humidity, the heat has significantly decreased, which has contributed to the improvement of the ecological quality of the area. The greenness of the “Ecology + Tourism” governance region increased after 2016, and the dryness and heat decreased, indicating that the vegetation in the area increased and the bare soil area decreased, thus the ecological quality of the area improved. The greenness index of the “Ecology + Poverty Alleviation” governance region is the highest, and the improvement of ecological quality in the area after 2016 is mainly attributed to the decrease of the dryness index, which indicates that the soil erosion in the area has been improved to a large extent.

### 4.4. Spatial Characteristics of Remote Sensing Ecological Indices in Different Governance Regions

Based on the classification of remote sensing ecological index, the ecological index patches of very poor, poor, medium, good, and excellent were considered as five landscape types, and the environmental ecological conditions of the four “Ecology+” governance regions were further analyzed from the perspective of the spatial distribution characteristics of remote sensing ecological index (Table 4).

The scale of patches with low ecological indices in the “Ecology + Industry” and “Ecology + Photovoltaic” governance regions is dominant. In the “Ecology + Photovoltaic” governance region, the connectivity of patches of the same level is stronger, and since the construction of the industrial park was gradually carried out in 2016, a very poor-grade ecological quality area with geometric contours was formed in the Shipai mining area. In the park, due to the positive effect of the Xunwu River and the planting of greenery in the park on the surrounding environment, the original poor ecological quality area with irregular boundaries was gradually divided. Also, in the “Ecology + Photovoltaic” management area, the same level of aggregation of patches is stronger. Take the part of photovoltaic power generation area located in the south of Hanshui District as an example; its map surface shows the transition from low ecological index to high ecological index in the form of circles or strips, the neighboring patches of the same grade are nested in a ring distribution, and small, highly aggregated patches of poor ecological quality are formed at the transition boundary.

The scale of patches with high ecological index dominates in the “Ecology + Tourism” and “Ecology + Poverty Alleviation” management areas. Take the “Ecology + Tourism” governance region located in the north of Hanshui District as an example; the medium and good ecological quality patches in the scenic tourism area are widely distributed and have a high degree of connectivity. However, there are still small patches of poor ecological quality scattered inside the tourist area. In the “Ecology + Poverty Alleviation” governance region, the patches of different grades are small in scale and show a more fragmented mosaic distribution.

## 5. Discussion

In this paper, we investigated the differences in ecological management effectiveness of the study area under the complex situation of two-stage and four-measure zoning management and the differences in ecological management effectiveness of the areas within the implementation of different stages and management means. The later zoning treatment is oriented to “sustainable regional economic and social development,” taking into account the location of the site, ecological substrate, and bare soil area of the mining area, and its treatment effectiveness is significantly better than that of the earlier treatment oriented to “plant regreening.”

The ecological quality of the area was further degraded during the three years from 2013 to 2016, as the area was not effectively covered by the remediation work of phased block management, and its poor ground conditions could not drive rapid natural recovery. The construction of industrial parks has led to an increase in construction land area, and the overall ecological quality of the region remained difficult to improve. However, from the perspective of Xunwu County, the construction of industrial parks has achieved the increase of green areas and the reduction of abandoned land in the county through the transposition of land resources, which has saved industrial land resources [45]. At the same time, the construction of the industrial park with ecological restoration as the main line, through the introduction of capital, quickly promotes the construction of green areas inside and around the park after the completion of the industrial park and plays a positive role in the ecological restoration of the region.

The “Ecology + Photovoltaic” governance region is based on a small area of bare soil with very poor ecological quality, by directly taking advantage of the abandoned mine's location in the countryside [46], open environment and its existing landscape with many height differences and flat terraces. This treatment method quickly eliminates the area of very poor quality grade in the region and achieves the transfer of abandoned mines to warehouse saving energy resources [47]. Although the area is still dominated by poor and very poor quality grade areas in the final area, the construction of the PV power station weakens the negative impact of the original mine on the surrounding ecological environment compared to the original bare earth mine.

“Ecology + Tourism” and “Ecology + Poverty Alleviation” are relatively more effective. The “Ecology + Tourism” governance region is relatively small in scale and flat in topography, and the ecological reconstruction and landscaping transformation is based on the depressed valley of the mine, thus rapidly and effectively improving the ecological quality of the area in a short time [48, 49]. The “Ecology + Poverty Alleviation” governance region is the most effective. This area has a good ecological foundation and is located in the less affected natural mountainous area to the north of the mining area, so the ecological spontaneous succession is faster after the mine stops mining. Agricultural means reduce the secondary damage to the site caused by guest soil restoration and chemical improvement [50] and at the same time promote local farmers to increase income while ecological restoration, taking into account the production value of the site, which provides a strong long-term drive for ecological restoration in the region and promotes the ecological transformation of the regional economic and social production methods and sustainable development [51].

There are complex dynamic feedbacks between land use type, land use structure, and ecological environment. A large number of studies have demonstrated the coupling relationship between landform and regional ecology based on the results of RSEI calculations and the concept of landscape ecology [52]. The differences in remotely sensed ecological indices formed by landscape patterns or landform characteristics in different regions can be reflected in the differences of different ecological quality patches in terms of area, shape complexity, spatial connectivity, and agglomeration [53]. In this study, the spatial distribution of ecological environment in the four “Eco+” management areas has different characteristics. In this study, the spatial distribution of the ecological environment in the four “Ecology+” management areas has different characteristics, which also indicates that the remote sensing ecological indices can reflect the ecological quality of the region at the macrolevel, and the spatial distribution of the remote sensing ecological indices can also be used to understand the landscape ecological pattern of the land surface.

The linear high ecological quality area in the “Ecology + Industry” governance region coincides with the location of Xunwu River. The higher quality ecological environment patches extending from Xunwu River to the east and west sides are related to the mountain flooding channel [54] transformed by the original deep ditch in the construction of the industrial park. In the “Ecology + Photovoltaic” governance region, with the help of RSEI's dryness index and heat index, the specificity of the metal material of photovoltaic cells is effectively reflected. The solar panels are arranged in a series on the sunny terraced site [55], left by mining to utilize the sunlight as efficiently as possible, thus forming a nested circle structure along the contour lines on the map. In the “Ecology + Tourism” and “Ecology + Poverty Alleviation” areas, the soil erosion is caused by engineering structures such as leaching ponds and tailing piles during mining, as well as the fragmented landforms such as multistage washouts and valleys within valleys are more fully expressed [56]. The small area of poor ecological quality patches in the “Ecology + Tourism” governance region is closely related to the broken terrain with many steep bumps and mountain cutoffs formed by mining. In the “Ecology + Poverty Alleviation” area, the economic forest and field planting area are relatively single and the RSEI index in the area mainly corresponds to the greenness index (NDVI) [57]. In order to avoid soil erosion aggravated by large volume of land remediation projects, vegetation and economic crops are often planted according to the topography of the terrain, and the measures such as replace out-soil backfill are taken in the reclaimed areas, so that the reclaimed areas show a mosaic distribution of small fragmented patches based on the original site. The mosaic structure of the agricultural landscape in previous studies is consistent [58].

However, only from the visualization results of RSEI, the ecological quality grade of the bare soil area caused by mining, the area where the new industrial park buildings are located, and the photovoltaic area where the new solar panels are laid are all extremely poor, and it is difficult to distinguish the effects of the three on the ecological quality of the area [59]. The reason is that the dryness index is obtained by inversion of the mean values of the building index and the bare soil index, which to a certain extent weakens the negative effect of the surface bare soil index on the ecological quality evaluation of rare earth mining areas and makes it difficult to intuitively reflect the regional impact of different features on the ecological environment. This study combined the idea of scale in landscape ecology and proposed the remote sensing ecological index based on moving window [60], which optimized the accuracy of the calculation of RSEI in the case of mixed regional feature types and was applicable to similar special cases such as the transfer of bare soil land and construction land in the mining area, and how to refine more targeted ecological evaluation indexes with this study needs further research.

## 6. Conclusion

In this study, the ecological restoration effect of mines in Xunwu County was evaluated based on the comprehensive remote sensing index RSEI, which found that

1. The absolute value of the contribution of the dryness index to the study area ranged from 0.65–0.81, which had the maximum contribution to the remote sensing ecological index of the study area. It indicates that the RSEI is applicable to the evaluation of mine ecological quality in areas with a large amount of bare soil. Meanwhile, the remote sensing ecological index comprehensively reflects the spatial and temporal differences in the effectiveness of treatment under the complex situation of two-stage and multi-instrument integrated treatment in the study area, which is applicable to the effectiveness assessment of the integrated ecological treatment of difficult standings.
2. The remote sensing ecological indices of the study area in 2013, 2016, and 2019 were 0.584, 0.613, and 0.667, respectively, with the mean value of the regional RSEI raised by 0.029 by the early fragmentation treatment, which is smaller than the mean value of the later RSEI raised by 0.051, reflecting the four “Ecology+” integrated management is more effective.
3. The areas with improved ecological quality are mainly located in tourism and poverty alleviation areas. The “Ecology + Poverty Alleviation” area has the highest ecological quality and the largest area of quality improvement and has the most positive effect on the improvement of the overall ecological environment in the study area, with a final RSEI value of 0.684; the “Ecology + Tourism” governance region has the largest degree of ecological quality improvement, with the largest increase in its remote sensing ecological index. The “Ecology + Tourism” governance region has the largest improvement in ecological quality, with an increase in the remote sensing ecological index of 0.244. The “Ecology + Photovoltaic” governance region has the smallest area and the worst ecological quality, but it has improved significantly compared with the beginning of the treatment, with an increase in the remote sensing ecological index of 0.163, and the area of the area with very poor ecological quality has decreased the most, with 46.53%. The overall ecological quality of the “Ecology + Industry” governance region is degraded, and the remote sensing ecological index decreases by 0.040, but the ecological degradation rate slows down at the later stage, and the area of the area with very good ecological quality grade increases by 1.75%.
4. The spatial distribution of remote sensing ecological indexes in the four “Ecological+” governance regions has different characteristics, which is of practical significance for the continued ecological construction and protection in each area in the later stage.

Throughout the study, because of the limitation of the revisit cycle of remote sensing satellites, it is difficult to unify the dates of the selected images when conducting ecological environment evaluation in the study area, which may have a certain impact on the comparability of the evaluation results. This study can also provide reference for the analysis of other regions and scales, and future related studies can further include the evaluation of natural resource occupation and ecological environment pollution in mines and carry out empirical investigation and research on the mechanism of the influence of related factors on the ecological environment of mines, so as to provide support for the construction of rural ecological civilization and rural revitalization.

## Figures and Tables

**Table 1:** Remote sensing ecological index calculation equation based on Landsat.

Year	Indicator	PC1	PC2	PC3	PC4
2013	NDVI	0.582089	0.574744	−0.39636	0.416822
	WET	0.455065	-0.451275	0.562854	0.521979
	NDBSI	−0.656506	0.039045	−0.126583	0.742599
	LST	−0.15195	0.681539	0.714192	−0.048427
	Eigenvalues	0.1271	0.0196	0.0074	0.0008
	Eigenvalue contribution rate	82.04%	12.64%	4.90%	0.52%

2016	NDVI	0.438591	0.403537	−0.625649	0.503348
	WET	0.33248	−0.212784	0.632208	0.666703
	NDBSI	−0.804157	−0.11432	−0.195237	0.549677
	LST	−0.224557	0.882503	0.41323	0.001794
	Eigenvalues	0.0899	0.0116	0.004	0.0006
	Eigenvalue contribution rate	84.69%	10.92%	3.78%	0.61%

2019	NDVI	0.600385	0.628438	−0.286336	0.403256
	WET	0.371651	−0.501933	0.51029	0.591222
	NDBSI	−0.66054	0.060444	−0.268693	0.698454
	LST	−0.255148	0.591165	0.765127	0.001885
	Eigenvalues	0.641	0.0076	0.0034	0.0004
	Eigenvalue contribution rate	84.91%	10.01%	4.51%	0.57%

**Table 2:** Area and proportion by grades of ecoenvironmental quality from 2013 to 2020.

Quality level	2013		2016		2019	
	Area (km^2^)	Proportion (%)	Area (km^2^)	Proportion (%)	Area (km^2^)	Proportion (%)
Very poor (0–0.2)	24.18	10.76	20.32	9.04	11.34868	5.05
Poor (0.2–0.4)	24.16	10.75	22.70	10.10	19.5512	8.70
Medium (0.4–0.6)	49.60	22.07	37.60	16.73	33.30445	14.82
Good (0.6–0.8)	85.82	38.19	97.35	43.32	91.48612	40.71
Very good (0.8–1.0)	40.97	18.23	46.74	20.80	69.01348	30.71
Mean	0.584199		0.613026		0.666642	
Total area, km^2^	224.7264					

**Table 3:** Remote sensing ecological index of different governance areas and overall area from 2013 to 2020.

	The whole area	Ecology + Industry	Ecology + Photovoltaic	Ecology + Tourism	Ecology + Poverty Alleviation
2013 RSEI	0.584199	0.396655	0.172896	0.368969	0.544972
2016 RSEI	0.613026	0.348723	0.205314	0.429426	0.607897
2019 RSEI	0.666642	0.359807	0.357332	0.621011	0.684398
Growth in value from 2013 to 2019	0.079977	−0.039912	0.163331	0.244082	0.138407
Area, km^2^	224.7264	73.6614	8.343	31.2093	111.5127

**Table 4:** Remote sensing ecological index performance of different management methods.

Governance area type	Patch composition characteristics	Patch distribution characteristics	Example
Ecology + Industry	Large area of poor ecological quality patches	Patches of the same grade have strong connectivity	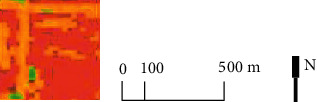

Ecology + Photovoltaic	Small area of poor ecological quality patches	Patches of the same grade were highly clustered, and patches of adjacent grades were distributed in circular nesting	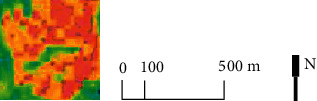

Ecology + Tourism	Large area of good ecological quality patches	Patches of the same grade have weak connectivity, the aggregation of patches with poor ecological quality was poor	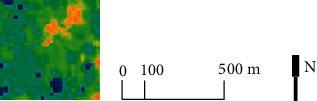

Ecology + Poverty Alleviation	Small area of good ecological quality patches	Patches of different grade fragmentation mosaic	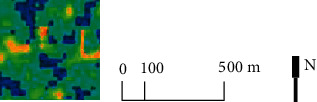

## Data Availability

The Landsat-8 data used to support the findings of this study are included within the article.
